# Trends of Parasuicide in Adolescents: A Hospital-Based Cross-Sectional Study

**DOI:** 10.7759/cureus.98193

**Published:** 2025-11-30

**Authors:** Kimi S Padhi, Manas Ranjan Sahu, Satyabrata Guru, Manoj Kumar Mohanty

**Affiliations:** 1 Forensic Medicine, All India Institute of Medical Sciences, Bhubaneswar, Bhubaneswar, IND; 2 Forensic Medicine and Toxicology, All India Institute of Medical Sciences, Bhubaneswar, Bhubaneswar, IND; 3 Trauma and Emergency Medicine, All India Institute of Medical Sciences, Bhubaneswar, Bhubaneswar, IND

**Keywords:** adolescent, attempted suicide, deliberate self-harm, parasuicide, risk factors of the suicide attempt

## Abstract

Background: "Parasuicide is referred to as an act with the nonfatal outcome, in which an individual deliberately initiates a nonhabitual behaviour that without intervention from others, will cause self-harm." Suicide is the leading cause of death among individuals aged 15-19 years. Because existing data indicate that non-fatal suicidal behaviour is the strongest predictor of suicide, the lack of non-fatal suicidal behaviour records presents a major gap in our understanding of the number of non-fatal cases and the dimensions of suicide.

Objective: To know the methods used in parasuicide and to identify the risk factors.

Method: A cross-sectional study was conducted at the casualty of AIIMS, Bhubaneswar. It included 105 adolescents who attempted parasuicide. Data were collected on various factors and trends associated with parasuicide and analysed.

Results: Adolescents aged between 15 and 19 years had a higher propensity for parasuicide. Gender-wise assessment showed that most of them were females (62.8%). The most commonly used method was self-poisoning (73.3%). Mass media exposure showed that mobile phones (69.5%) were one of the essential contributing factors. Academic issues (51.43%) were one of the common reasons behind the attempts. There was a slight decrease in the count of cases presenting to casualty with parasuicidal attempts during the COVID-19 pandemic. The various methods to attempt parasuicide used were significantly different in individuals with diverse academic backgrounds (p=0.04).

Conclusion: Parasuicide needs more focus and attention, which can be detrimental in preventing any future attempts of suicide.

## Introduction

Adolescence is a critical developmental period characterised by rapid biological, psychological, and social changes, during which young people may become vulnerable to emotional distress and self-harming behaviours [1]. Parasuicide is defined as an act with a non-fatal outcome where "an individual deliberately initiates a non-habitual behaviour that, without intervention from others, will cause self-harm," which is aimed at realizing changes which the subject desired, via the actual or expected physical consequences [2,3]. Self-inflicted injuries have always intrigued medical professionals as they are an important signal of underlying mental health problem and are recognised as a predictor of future suicide. According to global burden data for self-harm among adolescents aged 10-24 years in 2021, the incidence rate was approximately 89.64 per 100,000, and the mortality rate was approximately 5.93 per 100,000 [4]. Research indicates that self-harm occurs across all age groups, with lifetime prevalence rates estimated at approximately 3% in adults and 14% in children and adolescents [5,6]. However, notable gender differences exist in self-harm prevalence, with females exhibiting higher rates than males [4]. There is very little nationally representative data labelled "parasuicide" for Indian adolescents.

Although it is self-evident that one might attempt suicide before executing it, opinions suggest that suicide attempts do not always have death as their goal. Suicide intention is characterized by a varying spectrum of intensity/severity. It is affected by various (social, cultural, environmental, and spiritual) factors. The term "parasuicide" is sometimes used interchangeably with "attempted suicide" or considered to be the "lighter" version of attempted suicide (indicating little or no intention to die) [7]. Parasuicide affects life, temporarily or permanently, unlike suicide that terminates the life of an individual. Parasuicide is a problem of major concern in today’s society. The incidence of parasuicide is greatly influenced by differences in age, sex, race, religion, culture, marital status, habitat, climate, and social systems [2]. There is also a remarkable impact of natural disasters such as floods, cyclones, and droughts in the geographical area, as mentioned in one of the studies [8].The global pandemic situation is also expected to influence the trend of parasuicide.

This study aims to know the trends of methods used in parasuicide and identify modifiable and non-modifiable risk factors, through profiling that affect para-suicidal behaviour in the adolescent age group (10-19 years). The study includes the adolescent age group as young people just entering adolescence can face different challenges than those entering adulthood, which may be reflected in their self-harming behaviour. This age group lacks clarity in suicide ideation, self-harm, attempted suicide, and completed suicide. Therefore, all attempted suicide cases in the adolescent age group should be managed with the utmost attention.

## Materials and methods

This study was a cross-sectional observational study conducted in the Department of Forensic Medicine & Toxicology in collaboration with the Department of Trauma & Emergency at AIIMS, Bhubaneswar, from August 2019 to December 2020. This study was started after being approved by the Institutional Ethical Committee (IEC) of All India Institute of Medical Sciences, Bhubaneswar. The study population included all patients between the age group of 10 and 19 years with an alleged history of self-harm or attempted suicide reporting to the casualty of the Trauma & Emergency Department at AIIMS, Bhubaneswar. Participants were excluded from the study if they (1) had an unclear history, (2) were married, (3) were mentally disabled or differently abled, and (4) refused to give consent. The universal sample size method was adopted for the study population.

For each case in the study, psychiatric consultation was considered to confirm the study sample. Based on the diagnosis and clinical presentation, informed consent was obtained either from the patient or the relatives and guardians. Before filling out the proforma, the patient was again asked about the intent of the act, i.e., if they attempted the act with an intent to die or without an intent to die. Based on the reply, further information regarding the socio-demographic variables and the parasuicide-related indicators such as place of attempt, time of attempt, presence or absence of family members, stressor-related, any prior demand, type of method used, history of previous attempts, hesitation cut mark, use of mass media, academic performance, type of personality, addiction history, and details of the incident were obtained. After the data collection, the patient was followed up till the day of discharge and was counselled in each visit about the risks associated with the act and how to prevent this type of attempt in the future.

All collected data were entered in MS Excel 2019 (Microsoft Corp., Redmond, WA). The data were further analyzed using IBM SPSS Statistics for Windows, Version 22.0 (Released 2013; IBM Corp., Armonk, New York, United States). All categorical variables were expressed in terms of numbers and percentages. The association between two categorical variables was measured using the chi-square test. Quantitative variables were expressed in terms of mean and SD. A P-value of <0.05 was considered statistically significant. Correlation coefficient was derived to look for the strength of association between the various parameters, and logistic regression was performed to ascertain the effects of various parameters affecting the usage of various methods used in parasuicide.

## Results

This study was conducted in the casualty of AIIMS, Bhubaneswar, for 17 months from August 2019 to December 2020, and 105 individuals were selected as cases after meeting the inclusion criteria. After obtaining consent, a proforma was completed, which included various sociodemographic details, as presented in Table 1.

**Table 1:** Socio-demographic details of the study population. Data are presented as frequency (n) and percentage (%). Age is expressed as mean ± standard deviation (SD). Descriptive statistics were applied for analysis. Total study population: N = 105. APL: above poverty line; BPL: below poverty line.

Variable	Category	Frequency (n)	Percentage (%)	Mean ± SD
Age (in years)	10–14 years	29	27.61	13.7 ± 0.92
	15–19 years	76	72.39	17.5 ± 1.4
Sex	Female	66	62.86	—
	Male	39	37.14	—
Education	Primary	9	8.57	—
	Secondary	58	55.23	—
	Higher secondary	38	36.20	—
Academic performance	Average	51	48.57	—
	Good	20	19.05	—
	Poor	34	32.38	—
Socioeconomic status	APL	38	36.19	—
	BPL	67	63.81	—
Family type	Broken	2	1.90	—
	Joint	28	26.67	—
	Nuclear	75	71.43	—
Siblings	Present	101	96.20	—
	Absent	4	3.80	—
Personality	Sociable	89	84.76	—
	Stay alone	16	15.23	—

This depicts a higher prevalence of parasuicide in individuals aged between 15 and 19 years compared to individuals aged below 15 years. Females outnumbered males in attempting parasuicide. It was seen that low socioeconomic status and nuclear families had a higher prevalence of parasuicides. Seasonal variation when studied showed that 28 cases (26.66%) presented during the summer months (March-June), 38 cases (36.19%) during winter (November-February), and 39 cases (37.14%) during the rainy season (July-October).

Various methods were used by individuals included in the study, and it was observed that, out of all the methods, ingestion of poisonous substances was the most common method used to attempt parasuicide (77, 73.33%). Out of the 105 individuals, partially healed hesitation cuts were present in 24 (22.86%) of them, which gives knowledge of previous attempts. The most common motive behind an attempt was due to academic reasons, which was seen in 54 (51.42%) individuals, followed by love failures in 28 (26.66%). A significant number of individuals, i.e., 78 (74%), attempted the act indoors in the presence of family members. Cases during the pre-COVID months of the study period (July 2019-March 2020) were compared with the cases during the COVID pandemic months of the study period (April 2020-December 2020), and a higher proportion of cases were observed during the pre-COVID times.

Age- and gender-wise analyses were done, and individuals in the study were compared based on the substances they abused. A significant difference was found between the two groups. Academic performance was also compared with the substances abused, and it was found that higher prevalence was seen in individuals with poor academic performances. A chi-square test was performed, which showed a significant difference between the two groups (p=0.001) (Table 2).

**Table 2:** Relationship between socio-demographic and clinical variables with substance abuse among the study participants. Data are presented as frequencies (n), and p values were calculated using the χ² test. Statistically significant associations are indicated with an asterisk (*), and a p value of <0.05 was considered statistically significant. APL: above poverty line; BPL: below poverty line; χ²: chi-square.

Variable	Category	Substance abuse present (n)	Substance abuse absent (n)	χ² value	p value
Age (years)	10–14	4	20	6.05	0.014*
	15–19	18	63		
Sex	Male	20	19	10.85	0.001*
	Female	2	64		
Academic performance	Average	5	46	14.92	0.001*
	Good	0	20		
	Poor	17	17		
Socioeconomic status	APL	5	33	2.28	0.13
	BPL	17	50		

When the various demographic variables were compared to mass media exposure, it was found that mobile phone was the most commonly used mass media. The association was significant (p=0.04). Additionally, females who attempted parasuicide used the mobile phone more frequently, and the gender association was found to be significant (p=0.04).

The place of the attempt of parasuicide was also compared with age and gender, and it was found that both sexes preferred indoors over outdoors (p=0.006), but there was no significant difference found in the age groups compared.

The educational status and academic performance were compared with the methods used to attempt parasuicide. It was found that poisoning was the most common method used by individuals who completed secondary school education and had average academic performances. There was a significant difference between the various methods used and educational status (Table 3).

**Table 3:** Relationship between educational status, academic performance, and methods of attempt among participants. Data are shown as frequencies (n). p<0.05 was considered statistically significant. Statistically significant associations are indicated with an asterisk (*). χ²: chi-square.

Variable	Category	Poisoning (n)	Drug overdose (n)	Hanging (n)	Others (n)	χ² value	p value
Education	Primary	7	0	2	0	11.00	0.004*
	Secondary	44	8	2	4		
	Higher secondary	26	9	3	0		
Academic performance	Average	31	14	3	3	6.06	0.048*
	Good	16	2	2	0		
	Poor	30	1	2	1		
Total	—	77	17	7	4	—	—

Correlation of various parameters with the methods used in parasuicide (Table 4) was compared, and there was a significant negative correlation found between education status, socio-economic status, and substance abuse and the methods used in parasuicide. 

**Table 4:** Correlation between methods of parasuicide and various variables. Significant correlation at the 0.01 level (two-tailed) is indicated with double asterisks (**). p<0.05 was considered statistically significant (indicated with a single asterisk). r: Pearson’s correlation coefficient; χ²: derived chi-square value (approximation for effect size comparison).

Variable	r value	χ² value	p value
Age	0.658**	44.99	0.000*
Education status	-0.059	0.36	0.553
Mass media exposure	0.366**	13.75	0.000*
Family type	0.032	0.10	0.747
Socio-economic status	-0.284**	8.37	0.003*
Substance abuse	-0.327**	11.04	0.001*
Time interval	-0.098	1.00	0.320

The logistic regression model was statistically significant in cases of socio-economic status wherein a change from below poverty line (BPL) to above poverty line (APL) may use other means to commit parasuicide other than poisoning by 23.4%. In a patient with a previous attempt of parasuicide, there was 17% reduced chance of using poisoning as a method to attempt parasuicide (Table 5). 

**Table 5:** Regression analysis of factors associated with parasuicide. The regression coefficients (B), standard errors (S.E.), Wald statistics, degrees of freedom (df), significance levels (p values), odds ratios [Exp(B)], and 95% confidence intervals (CI) for Exp(B) are displayed. A p value of <0.05 was considered statistically significant.

Parameter	B	S.E.	Wald	df	Sig. (p value)	Exp(B)	95% CI for Exp(B)
							Lower
Socio-economic status	-1.454	0.637	5.199	1	0.023	0.234	0.067
Previous attempt	1.145	0.434	6.964	1	0.008	0.833	0.310

## Discussion

Parasuicide is preventable if we are identifying the factors contributing to it. Adolescence (10-19 years) is an age group where an individual attains puberty, and there are many hormonal changes. During this period, an individual’s overall mental and physical growth is expected to be in a certain way. Still, sometimes unexpectedly, there are specific abnormal presentations that may lead to parasuicide.

Childhood development of a person, exposure to various circumstances and situation plays a significant role. The changing education system, an evil competitive world, overenthusiastic parents, and peer pressure are one of the major reasons leading to parasuicide, and these factors are to be pondered. Along with the education system, there are other factors such as exposure to mass media like the Internet, which has contributed to a greater extent in disturbing the general pattern of behaviour and lifestyle. Therefore, the study was planned to identify the precipitating factor and know the trends among the adolescent age group and provide an insight on parasuicide.

In our study, the majority of individuals who attempted parasuicide were in the age group of 15-19 years, 76 cases (72.39%). This was consistent with studies conducted by Diggins et al. [9], Bharati et al. [10], Lingeswaran [11], and Griffin et al. [12] who stated that individuals less than 20 years of age were more vulnerable to attempting suicide or parasuicide and self-harm. Various studies have observed that the highest frequency of parasuicide is among females. This study has similar findings, with the ratio of female:male being 1.69:1. Females have less serious intent to die despite other findings illustrating no difference in suicide intent between males and females. Females often attempt suicide earlier in the evolution of psychiatric morbidity than males, representing less of an intention to die and more of a desire to communicate distress or change their social environment [13]. There was a difference in the age of occurrence of parasuicide, which can be attributed to the difference in their onset of puberty.

Academic performance plays a great role in the behavioural change of an individual. This was evident from the fact that 51% who were doing average in their academics attempted parasuicide. As per Kar [14], exam failure was considered a significant risk factor for suicide attempts, which is consistent with our study. In studies among the Mexican schoolgoing population, it was observed that there was an association between academic performance and attempted suicide, a prevalence of 3% among middle school students and 4.2% among high school students. It was postulated that the prevalence of attempted suicide increases with school years in students attending middle school, probably due to stress-related adjustments to adolescence [15]. It has been suggested in a study by Quiroga et al. [16] that some mental health problems, such as depression, are a risk factor for low academic performance. It was assessed in each of the individuals included in the study that parasuicides were more common in sociable people as a part of attention-seeking behaviour. This was similar to a study by Bi et al. [17] where people with high impulsivity and sociable traits had more serious attempts. In this study, around 76 (72%) of the patients had no history of any previous attempts, which was consistent with studies conducted on similar lines by Bharti et al.[10], Pradhan and Dhakal [18], and Al-Amin et al. [19]. Most of the patients, 77 (73.33%), adopted self-poisoning as the method, consistent with studies conducted by Arun et al. [20] and Narang et al. [21]. The reason for the dominance of organo-phosphorous compound poisoning as the method may be due to the easy availability and accessibility of these insecticides/pesticides to individuals. This result was consistent with findings from outside India, as reported by Diggins et al. [9] and Kim et al. [22], which were done on the adolescent population.

In our study, the poisoning was more common in the age group above 15 years than in individuals below 15 years, consistent with Diggins et al. [9]. The methods used were also compared and correlated to the individual's educational status, and it was found that around 41.9% who used poisoning as a method had at least secondary education. Drug overdose was seen in 17 (16.20%) of individuals, which was the second most common method used by individuals who attempted parasuicide, consistent with the findings by Halder and Mahato [23]. The more lethal methods were not commonly used because the intent to die was not there, as discussed in some of the studies conducted by Griffin et al. [12] and Al-Amin et al. [19]. Contrary to our findings found in a study by Pradhan and Dhakal [18], where the most common method employed to execute self-harm was hanging in males and drug overdose in females. In our study, drug overdose was compared between age groups, and it was seen that in the age group above 15 years, more individuals used drugs to attempt parasuicide, which was consistent with Griffin et al. [12]. There was a significant difference seen in the methods used by individuals with different educational backgrounds.

Substance abuse is very prevalent in India, and its usage is increasing in the younger adolescent population. Its association with suicidality is well known, as stated by Vijayakumar and Rajkumar [24]. In our study, there is a significant difference found between the sexes with respect to substance use. Adolescents with poor educational backgrounds and poor economic backgrounds were found to have a higher propensity for substance abuse. This was consistent with studies by Bharati et al. [10] and Pradhan and Dhakal [18]. There was a significant difference between the two age groups; adolescents older than 15 years were more prone to using illicit substances. Our study conducted a focused assessment comparing mass media exposure and found that mobile phone usage was the most common mass media in the 15- to 19-year age group, consistent with findings from Durkee et al. [25] and Shain [26], who reported that adolescents with Internet usage had higher suicidal ideations than non-mobile phone users. There was a significant difference between the age groups. The females who attempted parasuicide used mobile phones more as compared to males, and there was a significant difference between both sexes.

The COVID pandemic has affected the lives of people globally. However, parasuicide cases dropped, although not significantly, during these lockdown times. The emergency presentation has also reduced, and it was seen that the commonly used methods during the pre-COVID and COVID era were not different, i.e., poisoning was still the most common method used.

Limitations of the study

The study reflects the findings of patients attending tertiary care hospitals and may not be generalized to all parasuicide attempters in the general population. The sample size was small.

## Conclusions

Our findings of an increase in the rate of attempts among females in the 15- to 19-year age group are alarming and need further evaluation in future studies. The increased use of lethal self-harming methods among young people for non-suicidal reasons is a significant concern and needs immediate attention. The findings of this study have both clinical and practical implications that could help national policymakers and health providers identify susceptible groups in the future. Understanding parasuicide may help diagnose abnormal behavioural patterns, i.e., suicidal behaviour in the adolescent age group, which can be detected at an early stage. Therefore, parasuicide, which can be a transition stage between the intent to die, requires further understanding.
